# Surveillance, health promotion and control of Chagas disease in the Amazon Region - Medical attention in the Brazilian Amazon Region: a proposal

**DOI:** 10.1590/0074-02760150153

**Published:** 2015-11

**Authors:** José Rodrigues Coura, Angela CV Junqueira

**Affiliations:** Fundação Oswaldo Cruz, Instituto Oswaldo Cruz, Laboratório de Doenças Parasitárias, Rio de Janeiro, RJ, Brasil

**Keywords:** surveillance, health promotion, control, Chagas disease, Brazilian Amazon Region

## Abstract

We refer to Oswaldo Cruz's reports dating from 1913 about the necessities of a healthcare system for the Brazilian Amazon Region and about the journey of Carlos Chagas to 27 locations in this region and the measures that would need to be adopted. We discuss the risks of endemicity of Chagas disease in the Amazon Region. We recommend that epidemiological surveillance of Chagas disease in the Brazilian Amazon Region and Pan-Amazon region should be implemented through continuous monitoring of the human population that lives in the area, their housing, the environment and the presence of triatomines. The monitoring should be performed with periodic seroepidemiological surveys, semi-annual visits to homes by health agents and the training of malaria microscopists and healthcare technicians to identify *Trypanosoma cruzi* from patients' samples and *T. cruzi* infection rates among the triatomines caught. We recommend health promotion and control of Chagas disease through public health policies, especially through sanitary education regarding the risk factors for Chagas disease. Finally, we propose a healthcare system through base hospitals, intermediate-level units in the areas of the Brazilian Amazon Region and air transportation, considering the distances to be covered for medical care.

The Brazilian Amazon Region occupies 4,871,500 km^2^ (57.23% of Brazilian territory) and has the following characteristics: the largest fluvial complex in the world, the biggest sedimentary basin on the planet, the principal ecosystem of the biosphere, the greatest biodiversity of the planet and the leading potential for energy generation in Brazil. Thus, the region faces the difficult challenges in implementing a healthcare system that is effective in performing disease surveillance, control and providing care, especially because of the great distances that this region spans and the difficulty of access to services for the population. The Brazilian Amazon Region represents 67% of the area of the entire Pan-Amazon region (Castro 1998, Aguilar et al. 2007).

In a report to the Brazilian government dated 9 September 1913, Oswaldo Cruz recommended the construction of a central hospital and a research institute in Manaus, state of Amazonas (AM) health care facilities at the Madeira-Mamoré Railway and health units/hospitals in Coarí-Fonte Boa, in São Felipe by the Juruá River, in Vila Seabra by the Taraucá River, upstream along the Embira River and in Rio Branco, Abunã, Xajunrí and Porto do Acre, and proposed quinine therapy facilities in Boca do Acre, in Lábrea by the Purús River, in Sena Madureira by the Yaco River and in Santa Izabel by the Negro River. However, this plan is certainly now outdated.

In his conclusion, Oswaldo Cruz wrote: “If the plan for the sanitation campaign is implemented along the above lines, I can safely state that the capital obstacle that holds back the vertiginous progress to which the valley of the world's greatest river is destined will disappear and thus one of the richest, if not the very richest asset of Brazil, will be delivered to civilization. It is in the hands of the government to accomplish this.”.

Today, the Brazilian Amazon Region not only has the abovementioned challenges, but also has the following problems of paramount importance: (i) disorderly occupation, (ii) uncontrolled deforestation, (iii) risk of desertification and (iv) international covetousness. The Brazilian Amazon Region has borders with Bolivia, Peru, Equator, Colombia, Venezuela, Guyana, Suriname and French Guyana (Fig. 1), with a total length of over 16,886 km, which people cross accompanied by domestic animals. The great number of wild reservoirs and vectors infected by *Trypanosoma cruzi *makes Chagas disease hard to control.

**Fig. 1:**
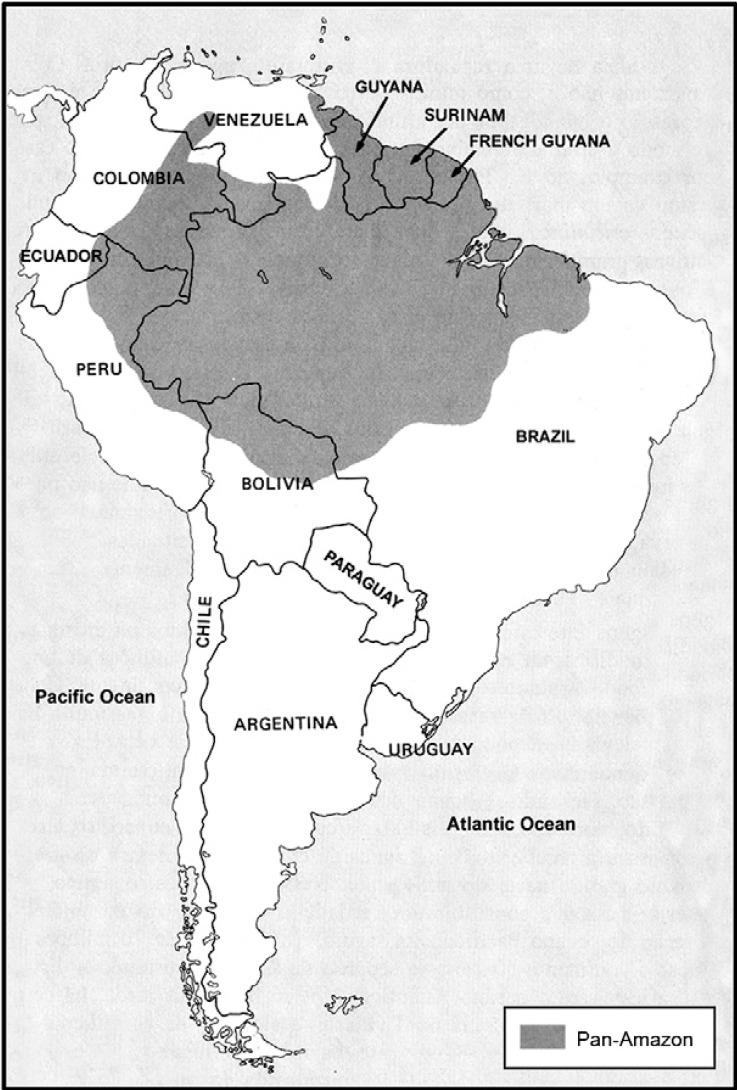
Pan-Amazon area in South America.

The risks of endemicity of Chagas disease in the Brazilian Amazon were highlighted over the past 20 years (Coura et al. 1993, 1994a, b,1995a, b, 1999, 2002a, b, 2013a, b,^2014^, Brum-Soares et al. 2010, Coura & Junqueira 2012, Coura 2013). These risks include the following. (i) The extensive reservoir of wild mammals, which consists of 33 species in six orders infected by *T. cruzi *(Coura & Junqueira 2012). Among the 27 species of triatomines identified in the Pan-Amazon region (Abad-Franch & Monteiro 2007), 16 were native to the Brazilian Amazon Region and 10 of these were reported to be infected with *T. cruzi*. (ii) The occurrences of various outbreaks of acute Chagas disease through oral transmission, since the end of the 1960s (Shaw et al. 1969, Valente & Valente 1993, Valente et al. 1999, 2009, 2009, Coura 2006, Pinto et al. 2008, Souza-Lima et al. 2013), involving over 1,500 patients in six states of the Brazilian Amazon Region. (iii) The uncontrolled deforestation of the Amazon Region, which has resulted in expulsion of the sylvatic animals of the area, which form the natural food source for triatomines, thereby stimulating their invasion into peridomestic and domestic areas in search of new sources of food. (iv) The increase in the migration of human populations from endemic areas accompanied by domestic animals infected with *T. cruzi* to the Amazon Region. (v) The lack of knowledge among the population of the Amazon Region regarding the risks of infection by *T. cruzi *through triatomines and wild mammals and, consequently, greater risk of exposure. Two indications of pre-adaptation of triatomines in the Brazilian Amazon Region are the presence of *Panstrongylus geniculatus*, found in pig-pens on the Marajó Island, state of Pará (PA) (Valente et al. 1998) and findings of *Triatoma maculata *in hen-houses in the rural area of the state of Roraima with occasional domestic incursions (Luitgards-Moura et al. 2005).

*Epidemiological surveillance* - Epidemiological surveillance of Chagas disease in the Brazilian Amazon Region, as well as in the Pan-Amazon region, needs to be performed through continuous study of the human population that lives in the area and its housing, identification of vectors (triatomines) and their presence or absence in peridomestic and sylvatic areas, rates of vector infection by *T. cruzi*, rates of vector infestation in the environment and the habits of the population with regard to invading the wild ecotope and the habits of wild reservoirs and vectors with regard to invading human domestic areas (Coura 2013, 2015, Coura & Junqueira 2015).

The human population that lives in the Amazon Region needs to periodically undergo seroepidemiological surveys in order to detect infection by *T. cruzi*, in general every three-five years. People involved in plant extraction activities, especially those who harvest piassava fibre, should be surveyed annually. Community agents should visit domestic and peridomestic areas (corrals, hen-houses and pig-pens) semi-annually, after being properly trained in how to survey for the presence of triatomines in mattresses, wall cracks, behind portraits, calendars, figures and posters inside the house, under rocks, debris and other hiding places in peridomestic areas, preferably using flashlights and after application of substances for dislodging triatomines and other insects as a routine of epidemiological investigation. When found, these insects should be placed in small containers such as empty matchboxes, with small holes in them for the insects to breathe, or in small cloth sacks or other receptacles. The agents should also write on the container or put a piece of paper in it, describing where the insect was found, the name of the person who is responsible for the home and the address of the location where it was found. These captured insects should be forwarded to the closest malaria diagnosis facility. The microscopists at these facilities have also been trained in accordance with the Training Manual for*Trypanosoma cruzi *Detection by Public-Service Malaria Microscopists and Laboratory Technicians (Coura et al. 2011), which has been widely distributed by the Health Public Laboratories of the Amazon Region.

The education departments of the municipalities of the Amazon Region should train elementary school teachers, through courses, to be able to guide their students in relation to triatomine surveillance. When students catch these insects, they should take them in a box to the school and the teacher should forward them to health agents, who will take the abovementioned actions.

The following species of wild triatomines have already been found in the Brazilian Amazon Region: *Belminus herreri*, *Cavernicola lenti*,*Cavernicola pilosa*, *Eratyrus mucronatus*,*Microtriatoma trinidadensis*, *P. geniculatus*,*Panstrongylus lignarius*, *Panstrongylus anfotubercutalus*, *Rhodnius brethesi*, *Rhodnius nasutus*, *Rhodnius neglectus*, *Rhodnius paraenses*, *Rhodnius pietipes*, *Rhodnius robustus*, *T. maculata* and *Triatoma rubrofasciata* (Coura et al. 2002b). Most of these species live in palm trees and feed on the blood of wild birds and/or mammals. Table shows which of these species have been found infected with *T. cruzi*. In turn, 33 species of mammals of the orders Carnivora, Chiroptera, Edentata (Xenarthra), Marsupialia (Didelphimorphia) and Primates, in the Amazon Region, have also been found infected by *T. cruzi *(Coura & Junqueira 2012). Thus, the surveillance of Chagas disease in this area should be aimed towards the abovementioned species of triatomines and orders of mammals, particularly in palm trees close to homes, to which adult triatomines fly and then invade homes, as well as some mammals such as marsupials, which may enter homes seeking food and contaminate it with the secretions from their odour glands.

**TABLE:** Triatomines found in the Brazilian Amazon


*Belmirus herreri*
*Rhodnius brethesia*
*Cavernicola lenti*
*Rhodnius nasutus*
*Cavernicola pilosa*
*Rhodhnius neglectus* ^*a*^
*Eratyrus mucronatus* ^*a*^
*Rhodnius paraensis* ^*a*^
*Microtriatoma trinidadensis* ^*a*^
*Rhodnius pictipies* ^*a*^
*Panstrongylus geniculatus* ^*a*^
*Rhodnius robustus* ^*a*^
*Pantrongylus liguinarius* ^*a*^
*Triatoma maculata*
*Pantrongylus rubotuberculatus* ^*a*^
*Triatoma rubrofasciata*

*a*: infected with *Trypanosoma cruzi*. Apud Coura et al. (2002b).

Oral transmission of *T. cruzi* is now the most frequent transmission mechanism for the parasite, through food contaminated with the faeces and urine of triatomines or with the odorous secretions of marsupials, not only in the Amazon Region, but also in other regions of Brazil. The first description of an outbreak of acute Chagas disease probably transmitted in this manner in Brazil was made by Silva et al. (1968) and Nery-Guimarães et al. (1968) without determining the source of infection. The outbreak occurred in Teutônia, in the municipality of Estrela, state of Rio Grande do Sul and involved 18 people, of whom six died. In October 1986, another acute outbreak occurred in the municipality of Catolé do Rocha, state of Paraíba (Shikanai-Yasuda et al. 1991), in which the source of infection was probably sugarcane juice. It involved 26 people with one fatal case. Other poorly defined outbreaks occurred in the states of Ceará and Bahia. Another similar one occurred in 2005, in the municipality of Navegantes, state of Santa Catarina, with 19 cases and three deaths (Steindel et al. 2008). The first four cases in the municipality of Belém, PA, were described by Shaw et al. (1969). Since then, many cases have occurred in this region and most of them were attributed, through inference, to consumption of *açaí* juice because all the patients drank juice from the same source, especially in the states of Amapá, Maranhão, Acre, AM and PA (Valente et al. 1999, 2009, Medeiros et al. 2008,Pinto et al. 2008, Barbosa-Ferreira et al. 2010, Souza-Lima et al. 2013). The surveillance of transmission cases is extremely hard because such cases are unpredictable. However, from the index case (the 1st one that was diagnosed) other cases that were infected from the same food source can be located through direct blood examination and findings of *T. cruzi *in symptomatic and asymptomatic patients. Four South American countries have notified outbreaks that were probably orally transmitted: Bolivia (1), Venezuela (5), Colombia (6) and Brazil (> 70). The largest outbreak was described in Caracas, Venezuela, with 103 cases (Alarcon de Noya et al. 2010, 2015). This outbreak occurred in 2007. Since then, 10 other outbreaks (4 of them in Caracas) consisting of 249 cases (23.5% in children) with 4% mortality. The highest number of outbreaks of Chagas disease since Shaw et al. described the first one in 1969 has occurred in the Brazilian Amazon Region (Coura & Junqueira 2012, 2, 2015).

*Health promotion and disease control* - Health promotion has been defined by the World Health Organization (WHO) as “the process of enabling people to increase control over and to improve, their health”. The forms of health promotion occur through public health policies, for which financial resources, housing quality and food and work security are prerequisites (WHO 2005). More recently, there has been a tendency among governments and public health officials, especially in the liberal nations such as Canada and the United States of America, towards reducing health promotion and education in favour of social “marketing” focused on behavioural change regarding risk factors. In this context, health is a positive concept of emphasising social and personal conditions, as well as physical ability (WHO 1986).

In the case of Chagas disease in the Amazon Region, the use of education for promoting health should be followed, thus avoiding the risks of Chagas disease among the resident population and improving health, through the following actions: (i) to educate the population in order to keep it as far as possible from the reservoirs and wild vectors, thus avoiding the presence of palm trees next to homes, as well as corrals, pig-pens and hen-houses, (ii) to wash fruits before eating them, to cover blenders of*açaí* and other fruit juices, which also should be washed before blending and to avoid eating raw meat from wild animals, (iii) to avoid unnecessary deforestation, except near homes, (iv) to catch triatomines that may enter homes and take them to the local health agents so that these insects can be forwarded for examination and (v) to get information for themselves and stimulate family members to become informed about the risks of contracting Chagas disease and how to avoid it.

The prospects for controlling Chagas disease in the Amazon Region are very limited because the majority of acute cases are caused by orally transmitted outbreaks (Pinto et al. 2008). Vector transmission is almost always related to people involved in plant extraction activities, especially piassaba harvesters (Coura et al. 2002a, b, Brum-Soares et al. 2010), or occurs accidentally through people's invasion of the wild ecotope for hunting, fishing or other activities, or when wild animals (marsupials) and vectors infected with *T. cruzi *invade human homes seeking food or, in the case of vectors, attracted by light. Considering these factors, the methods for controlling Chagas disease in the Amazon Region are necessarily different from the techniques applied in endemic areas, where the vectors are domesticated and the transmission occurs continuously (Dias et al. 2002, Coura & Dias 2009). The means for controlling Chagas disease in the Amazon Region is predominantly related to educating the population and healthcare professionals, such as technicians in laboratories for diagnosing malaria, who need to be trained to diagnose *T. cruzi* infection and the agents of endemic diseases so as to search for *T. cruzi* and identify triatomines. Likewise, doctors and nurses need training in order to treat the disease.

*Medical attention in the Brazilian Amazon Region: a proposal - *Over 100 years ago, Oswaldo Cruz made a proposal for a health care system for the Brazilian Amazon Region, which became outdated without ever being implemented. Some actions were taken by Oswaldo Cruz himself (1910-1912), when he introduced sanitation measures on the Madeira-Mamoré Railway (malaria control), yellow fever control in Belém and the sanitation system of Manaus. Between 1912-1913, Carlos Chagas made a long journey and reported on visits to 27 different locations in the Amazon Region, from which he detailed the sanitation conditions. In this report, he mentioned the sanitation conditions of Manaus and the regions of the Solimões, Juruá, Purús, Tarauacá, Acre, Branco and Negro rivers.

This interest in the Brazilian Amazon has been continued by research workers from his institute. Olympio da Fonseca Filho, installed the National Institute of Amazon Research, which was visited by various researchers from the Oswaldo Cruz Institute (IOC) between 1956-1958. In 1994, the Oswaldo Cruz Foundation established a Technical Office in Manaus, which was transformed into the Leônidas and Maria Deane Research Centre in 2001. In 1991, our Parasitic Diseases Laboratory at the IOC established an extension in the municipality of Barcelos, beside the Negro River, where several research projects and theses on viral hepatitis, Chagas disease, malaria and helminthiasis have been developed.

Healthcare in the Brazilian Amazon Region, starting in 1956, was partly attended by the National Department of Endemic Rural Diseases, the Special Public Health Service (SESP) and the Superintendency of Public Health Campaigns. These were later transformed into the National Health Foundation. Currently healthcare is provided by the Brazilian National Health System, with different levels of activity.

The proposal for medical care in the Brazilian Amazon Region that we made to the Amazon Surveillance and Protection System (SIVAM-SIPAM), by invitation from the SIVAM organisers, consisted of eight Base Hospitals, more than 35 SESP-type intermediate units with one clinical doctor, one surgeon, one paediatrician, one gynaecologist-obstetrician, one dentist, one graduate nurse and assistants for each unit and mobile units with air transportation (ship hospital with helicopter to transport the patients from remote areas to the health units) (Fig. 2). For unknown reasons, SIPAM never implemented the healthcare system, although it is entirely dedicated (so we hope) to the security of the Brazilian Amazon Region.

**Fig. 2:**
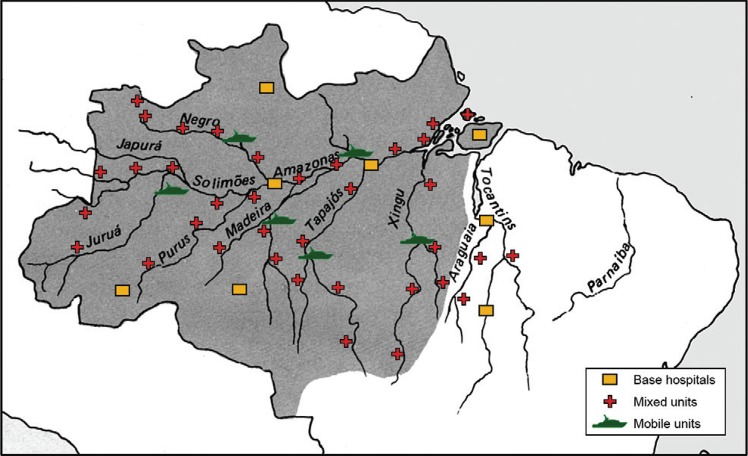
a proposal for medical attention in the Brazilian Amazon.
